# IgG4-related Mastitis: An Underrecognized Diagnosis in Breast Core Biopsies

**DOI:** 10.5041/RMMJ.10578

**Published:** 2026-04-26

**Authors:** Payam S. Pahlavan

**Affiliations:** Department of Pathology, Lutheran Hospital, Fort Wayne, Indiana, USA

**Keywords:** Core needle biopsy, IgG4-related mastitis, underdiagnosis

**To the Editor**,

We read with great interest the comprehensive review article, “IgG4-related Breast Disease: Review of the Literature,” by Jeries et al., published in the October 2024 issue of *Rambam Maimonides Medical Journal*.^1^ This timely synthesis highlights the critical challenge posed by immunoglobulin G4-related breast disease (IgG4-BD), a rare, tumor-like manifestation of a systemic condition that frequently presents as a lump or mastitis. We fully concur with the authors’ findings but wish to further emphasize the concern that isolated IgG4-related mastitis (IgG4-RM) may be underreported and significantly underdiagnosed in routine practice, particularly when evaluation is restricted to analysis of breast core needle biopsies (CNBs).

## RATIONALE FOR HEIGHTENED SUSPICION

The clinical scenario necessitating biopsy is almost universally concerning: a firm, palpable mass often discovered via routine mammography or breast ultrasound. In the modern diagnostic algorithm, a CNB is the indispensable, standard-of-care procedure for evaluating all suspicious breast findings. While the initial and primary goal of pathological review is to swiftly and accurately rule out malignancy, the detailed sub-categorization of benign lesions, or the provision of a thorough differential diagnosis in the context of limited tissue, is crucial because it directly dictates the appropriate clinical management path. Immunoglobulin G4-related mastitis is one such entity where an accurate benign diagnosis prevents inappropriate, morbid treatment.

## PATHOLOGICAL AND DIAGNOSTIC CHALLENGES IN CNB

Immunoglobulin G4-related mastitis is a histopathological chameleon. Isolated IgG4-RM is classically defined by a histopathologic triad:^2^ (1) storiform stromal fibrosis; (2) a dense lymphoplasmacytic infiltrate with a high proportion of IgG4-positive plasma cells, and (3) obliterative phlebitis.

However, in IgG4-BD, Jeries et al. noted that the presence of the complete triad is rare, with only 31.4% of the analyzed cases exhibiting all “classic” signs.^1^ This incomplete presentation creates significant diagnostic difficulties when reviewing CNB samples for several reasons:

- *Non-specific overlap with differential diagnoses:* The basic histological features of fibrosis and dense plasma cell infiltration are non-specific, causing significant overlap with several key differential diagnoses that often lead to misclassification. These include plasma cell mastitis (which also features dense plasma cell infiltration), granulomatous lobular mastitis (a chronic inflammatory condition), inflammatory pseudotumor, and even mucosa-associated lymphoid tissue (MALT) lymphoma.^2^,^3^ Differentiating IgG4-RM from these entities, especially MALT lymphoma, is highly challenging and requires a high index of suspicion.
- *Tissue sampling limitations:* By nature, CNBs sample only small columns of breast tissue. This limited sampling frequently fails to capture the entire classic triad, particularly the key, but less common, feature of obliterative phlebitis. Without a suggestive clinical history or a request for special stains, the diagnosis will often default to the less specific category of “chronic inflammatory mastitis.”
- *Need for IgG4 immunohistochemistry:* The definitive diagnosis hinges on the application of immunohistochemical staining to confirm the plasmacytic phenotype. The criteria require more than 10 IgG4-positive plasma cells per high-power field and an IgG4+/IgG+ plasma cell ratio exceeding 40%.^4^ Crucially, this supplementary staining is not routinely ordered for samples initially ruled out for malignancy. This leads to the under-prioritization and missed diagnosis of IgG4-RM, particularly in smaller pathology practices where the lack of in-house IgG4 immunohistochemistry may necessitate ordering it from outside centers, adding to cost and turnaround time and thereby further restricting its use.

## RADIOLOGICAL AND CLINICAL MIMICRY

The clinical presentation of IgG4-RM further complicates the diagnostic path, as it frequently mimics advanced malignancy. It typically presents as a tumor-like mass, sometimes accompanied by features such as skin thickening or axillary lymphadenopathy,^3^,^5^ leading to a high clinical suspicion of breast cancer or inflammatory breast cancer.

Radiologically, however, a subtle yet pivotal clue often exists. Unlike typical malignant masses, which frequently contain microcalcifications and present with spiculation or irregular margins, the radiological features of IgG4-RM are less aggressive. A key radiological sign is the typical absence of calcifications on ultrasound, mammography, or computed tomography.^6^ Recognizing the triad of a suspicious clinical presentation, non-diagnostic CNB (benign inflammatory cells), and the absence of calcifications should immediately raise the pathologist’s index of suspicion and prompt ordering the necessary IgG4 stains.

## CLINICAL SIGNIFICANCE AND MANAGEMENT

The clinical significance of an accurate pathological diagnosis is profound, as it holds major therapeutic and prognostic consequences for the patient. Unlike malignancy, IgG4-RM is a medically managed disease that does not require invasive surgical excision (lumpectomy or mastectomy). An erroneous diagnosis resulting from inadequate pathological work-up leads to unnecessary, costly, and morbid surgery. Instead, the standard first-line treatment for IgG4-RM is systemic corticosteroids (e.g. prednisolone) to induce clinical and radiological remission.^3^,^6^ Emerging evidence also suggests that some stable cases may even be managed with careful clinical and imaging follow-up alone, without the need for steroids or excision.^7^ For refractory disease, advanced immunosuppression with anti-CD20 biological therapy (e.g. rituximab) has been successfully employed.^8^ Therefore, an accurate diagnosis provides essential reassurance to the patient by definitively excluding malignancy and guiding them toward the correct non-surgical care. Table 1 summarizes the key comparison features of IgG4-RM versus malignancy.

**Table 1:** Clinical Outcomes and Management Implications.

Clinical Presentation and Management Aspects	Breast Cancer (Malignancy)	IgG4-related Mastitis (IgG4-RM)
Initial presentation	Firm mass, skin changes, lymphadenopathy	Firm mass, skin thickening, lymphadenopathy (mimics cancer)
Definitive treatment	Surgical excision (lumpectomy/ mastectomy), chemotherapy, radiation	Systemic corticosteroids (prednisolone) to induce remission
Novel/alternative treatment	Targeted therapy, immunotherapy	Advanced immunosuppression (e.g. rituximab), or careful observation in stable cases

## CALL TO ACTION

Given the potential for this benign but clinically and radiologically significant condition to be mistaken for malignancy or other complex entities, we hypothesize that the true incidence of isolated IgG4-RM in breast is underreported. This underdiagnosis may be attributed to the limitations of CNB tissue sampling, unfamiliarity with the entity, and the restricted use of IgG4 staining. We therefore urge breast pathologists to maintain a high index of suspicion for IgG4-RM. When reviewing CNBs that demonstrate a fibrosclerotic stroma with a prominent lymphoplasmacytic infiltrate, ordering supplementary IgG4 staining is a relevant and necessary step toward achieving accurate diagnosis and guiding appropriate non-surgical patient management. This proactive approach is essential to increasing the awareness and diagnosis of IgG4-BD, as suggested by Jeries et al.^1^

## Abbreviations

CNB: core needle biopsy
IgG4: immunoglobulin G4
IgG4-BD: immunoglobulin G4-related breast disease
IgG4-RM: immunoglobulin G4-related mastitis
